# Prognostic Biomarker NUMB Is Inhibited by Breast Cancer Cell Exosomes to Promote Breast Cancer Progression

**DOI:** 10.1155/2022/6032076

**Published:** 2022-04-18

**Authors:** Xue Qin, Youde Cao

**Affiliations:** School of Basic Medical Sciences, Chongqing Medical University, Chongqing 400016, China

## Abstract

**Objective:**

To clarify the regulation of breast cancer cell-derived exosomes on breast cancer and the expression of the NUMB endocytic adaptor protein (NUMB) protein.

**Methods:**

The exosomes of breast cancer cell line MDA-MB-231 were isolated. The exosomes were subsequently labeled with PKH67 and added to breast cancer MDA-MB-231 cells cultured *in vitro*. Transwell and clone formation assays were performed to detect cell migration, invasion, and clone formation. Meanwhile, Western blot and qPCR were conducted to determine the regulation of NUMB expression by exosomes in breast cancer cells. Furthermore, NUMB overexpressed lentivirus was supplemented to validate the recovery.

**Results:**

The number of migrating and invasive breast cancer cells in the exosome-treated group was significantly increased compared with the control group. Moreover, the number of breast cancer cell clones in the exosome-treated group was increased than in the control group. However, the NUMB expression in breast cancer cells treated with exosomes revealed a substantial decrease, indicating that the exosomes of breast cancer cells could inhibit NUMB expression. NUMB overexpressed lentivirus supplementation markedly suppressed cell migration, invasion, and proliferation of breast cancer cells compared with exosome group.

**Conclusion:**

Taken together, the exosomes of breast cancer cells could inhibit the expression of NUMB and promote the migration, invasion, and cell clone formation of breast cancer cells.

## 1. Introduction

The capacity of reversible exchange between different cellular states is cancer cell plasticity that includes a mutual transformation of several subtypes of cancer cells, activation, transdifferentiation, or dedifferentiation of facultative cancer stem cells, and phenotypic transformation of differential cells in a tumor to meet challenges of a microenvironment and therapeutic intervention [1–3]. Breast cancer represents the top one serious threat to females among all types of cancers, resulting in approximately 500,000 deaths annually. As a complex and heterogeneous cancer, it varies greatly from patient to patient and from tumor to tumor (intratumoral heterogeneity) [4]. Altering cellular plasticity can promote the occurrence of breast cancer cell heterogeneity and enhance cancer cell proliferation, migration, and tolerance [5].

The plasticity of cancer cells can be induced by exosomes, which are mainly derived from the vesicles. Studies have reported that exosomes can affect the plasticity of cancer stem cells by mediating communication between tumor cells and their microenvironment, activating the Notch3 signaling stem pathway in breast cancer cells, and inducing epithelial-mesenchymal transition (EMT) [6–8]. The vesicles release inner vesicles in the form of exosomes by fusing with the plasma membrane. Exosomes are enclosed by a lipid bilayer [9]. Almost all types of cells in the body can release exosomes. Electron microscopy results indicate that they have disk- or cup-like structures, with diameters ranging from 30 to 150 nanometers. With the advantages of size, density, shape, and surface protein, exosomes can be separated by ultrafast centrifugation, ultrafiltration, and immunoprecipitation [10]. The content of exosomes may vary from cell source, activation state, and cell type. Meanwhile, they contain certain special contents, proteins associated with membrane transport and fusion (flotillin, GTPases, and annexins), multivehicle biogenesis (TSG101 or Alix), tetrapeptide family proteins (CD9, CD82, CD81, and CD63), heat shock proteins (CCT2, HSPA5, Hsp90, HSP70, and HSP60), and cell apoptosis related lipid-binding proteins [3–11]. Breast cancer cells communicate with each other through direct or indirect contact, namely, exosomes secretion to adapt to changes in condition [12]. As a result, glucose concentration decreases while acidity increases, which in turn inhibits immune cell infiltration, leading to tumor immune escape and tumor invasion [13].

The NUMB endocytic adaptor (NUMB) protein is of great significance in the occurrence and development of breast cancer. Some research has demonstrated that the expression of NUMB alters p53 levels in breast cancer [14, 15]. The link of NUMB to a cancer stem cell phenotype was demonstrated, and the loss of NUMB expression is a marker of tumor aggressiveness in primary breast cancer [16]. Meanwhile, it is positively correlated with the classification and prognosis of breast cancer [17, 18]. The present study was aimed at figuring out whether exosomes have any effects on migration and invasion of breast cancer cells and whether NUMB protein produces any regulatory roles or not. In this study, isolated exosomes of breast cancer cells were added to breast cancer cells *in vitro* for detection and the role and molecular mechanism of exosomes in the treatment of breast cancer were subsequently explored.

## 2. Materials and Methods

### 2.1. Isolation of Exosomes from Breast Cancer Cells

MDA-MB-231 breast cancer cell line was purchased from American Type Culture Collection (HTB-26, ATCC, USA). Breast cancer cells were inoculated into a culture dish by adding Dulbecco's Modified Eagle Medium/Nutrient Mixture F-12 (DMEM/F12) medium (Hyclone, USA) containing 10% exosomes-free serum (A27208-03, Gibco, USA) and 1% penicillin/streptomycin and cultured in a 37°C, 5% CO_2_ incubator. To isolate the exosomes, the medium was removed by centrifugation at 10,000 g/min 4°C for 30 min, the sediment was collected and precipitated using PBS (pH = 7.0), and then the exosomes were extracted using an exosome column (ThermoFisher, USA) [19]. An equal volume of phosphate buffered saline (PBS) was used as a control group.

### 2.2. NUMB Overexpression Lentivirus Packaging and Cell Transfection

NUMB overexpression lentivirus was constructed and packaged by Chongqing Biomedicine Biotechnology Co., Ltd. Cell transfection was performed using Lipofectamine2000 kit (Invitrogen, USA). Cells were collected 24 hours after transfection for testing.

### 2.3. Characterization of the Exosomes by Electron Microscope and Western Blot

Exosomes were added with 2.5% glutaraldehyde, incubated on a transmission electron microscope grid at room temperature for 2 min, supplied with 3% phosphotungstic acid (Sangon Biotech, China), and left undisturbed at room temperature for 2 min. Exosomes were observed under the microscope and photographed (Mshot M073, Guangzhou, China).

Protein expression levels of CD9 and CD63 were detected by WB. Exosomes were denatured at 95°C in SDS buffer solution for 10 min. Following separation using gel electrophoresis with 10% sodium lauryl sulfate polyacrylamide (SDS-PAGE), the protein was transferred to a polyvinylidene difluoroethylene (PVDF) membrane, sealed with skimmed milk powder for 2 h, incubated overnight by adding anti-CD9 antibody and anti-CD63 antibody (Abclonal, China) at 4°C. The anti-rat IgG antibody (Abclonal, China) was coupled with horseradish peroxidase. The gray value of the final protein band was analyzed with Image J (Rawak Software Inc., Stuttgart, Germany) [20, 21].

### 2.4. PKH67-Labeled Exosomes

The amount of exosome protein was determined by the BCA (Beyotime, China) method. 50 mL PKH67 dye working solution (ThermoFisher, USA) was added to every 100 micrograms of protein and then incubated for 10 min at room temperature. After the mixture was mixed with 10 mL PBS, the exosomes were isolated from the mixture and purified with 200 *s*L PBS. The purified exosomes were added to cultured breast cancer cells at the ratio of 20 *μ*L exosome/mL cell medium for 24 h [22, 23].

### 2.5. Detection of Cell Migration and Invasion Using Transwell Assays

The MDA-MB-231 cells were cultured until the logarithmic growth phase before measuring, followed by digestion, one cycle of washing with PBS, and serum-free medium and suspended with serum-free medium. The cell count was subsequently calculated and adjusted at the concentration of 2 × 10^5^ cells/mL. During the invasion experiment, matrigel (ThermoFisher, USA) glue was stored in a -20°C condition and then placed in a refrigerator at 4°C overnight. Matrigel glue was then diluted using a serum-free medium at a concentration of 300 *μ*L/mL, and 100 *μ*L was taken and evenly smeared on the upper surface of the PET membrane of a cell culture pool. Then, the pool was gently placed into a well of a 24-well plate, kept at 37°C for about 3 h, taken out, and dried overnight on a clean table. Following the addition of 600 *μ*L medium with 10% serum in the lower chamber and 150 *μ*L cell suspension in the upper chamber, the cells were treated with the ratio of 20 *μ*L exosomes/mL cell medium (the control group was equal volume PBS) for 24 h. The lower surface was soaked in a 70% methanol solution, fixed for 30 min, stained with crystal violet, and visualized under a microscope, and the number of cells on the lower surface of the PET film was calculated.

### 2.6. Plate Cell Cloning and Formation Test

At the logarithmic growth phase, the MDA-MB-231 cells of all experimental groups were digested using 0.25% trypsin (Beyotime, China), blown to single cells, and suspended in a DMEM medium of 10% fetal bovine serum for later experiments. 300 cells were seeded into a dish containing 10 mL of preheated culture solution at 37°C and gently shaken to make the cells evenly dispersed. The cells were inoculated in an incubator at 37°C with 5% CO_2_ and treated with the ratio of 20 *μ*L exosomes/mL cell medium. The culture was terminated after 1 week. Following, the supernatant was discarded and the cells were soaked in PBS carefully twice. Subsequently, the cells were fixed for 15 min using 4% paraformaldehyde (Sangon Biotech, China). The fixed solution was removed, and the crystal violet was dyed for 10 min, then the dyeing solution was slowly washed away with running water and dried in air.

### 2.7. Detection of NUMB mRNA Expression by RT-PCR

After being washed with PBS, the cells were added with Trizol (Invitrogen, China), and the lysate was transferred to a new EP tube, which was added with 200 *μ*L precooled chloroform to each 1 mL Trizol, iced for 5 min, centrifuged at 4°C for 15 min, then carefully sucked out the supernatant into a new EP tube. Precooled anhydrous ethanol was supplied, mixed well, and left undisturbed. The RNA was precipitated by centrifugation at 4°C. The supernatant was discarded, and 75% ethanol was added and centrifuged at 4°C. Following the disposal of the supernatant completely, and diethyl pyrocarbonate (DEPC) was supplied to dissolve after standing. The supernatant was removed completely. DEPC water was added after standing, dissolved, mixed well, and kept 1 *μ*L for quantification and electrophoresis. Data processing and analysis were carried out for histogram plotting. The primer sequence was as follows: NUMB-F, TGAGCCAACTGTGTCAGGAG; NUMB-R, AATGCTGCGATTGTTGTTGATGF; GADPH-F, GGCACAGTCAAGGCTGAGAATG; and GAPDH-R, ATGGTGGTGAAGACGCCAGTA [24].

### 2.8. Detection of NUMB Protein Expression by Western Blot

Frozen supernatant was taken out for protein quantification. SDS-PAGE gels with 10% and 5% SDS denaturation were prepared. Protein samples were mixed with loading buffer for electrophoresis. Total proteins were electrophoretically transferred to PVDF membranes and then continuously incubated with primary and secondary antibodies corresponding to NUMB (1 : 1 000) that is purchased from Abcam Trading (Shanghai) Co., Ltd (ab264224). The film was placed in a chromogenic agent, exposed, developed, and fixed. Image J was used to analyze the gray value of scanning results.

### 2.9. Statistical Analysis

Statistical analysis and mapping were performed utilizing GraphPad Prism 8.0 (GraphPad Software Inc., USA), and *t*-tests were adopted for pairwise comparison. # *p* < 0.05 was considered statistically significant, and ## *p* < 0.01 was considered extremely significant.

## 3. Results

### 3.1. Detection and Identification of Breast Cancer Exosomes

**Figure 1:**
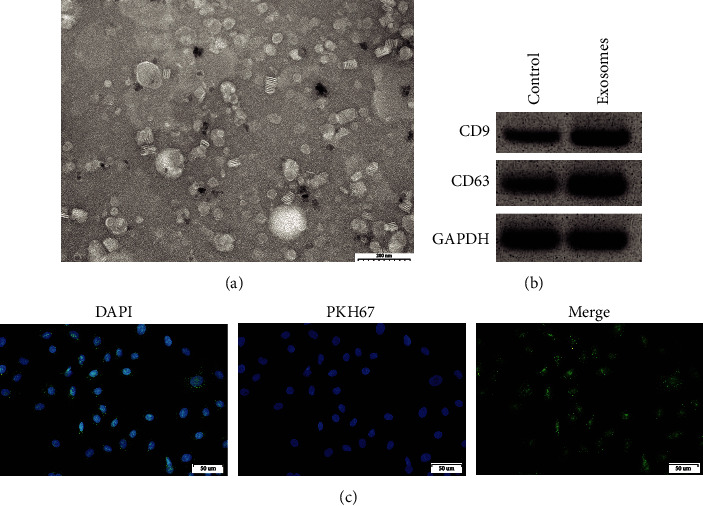
Isolation and identification of exosomes from MDA-MB-231 breast cancer cells. (a). Observation of the MDA-MB-231-exosome structure under electron microscopy (scale bar: 200 nm); (b). Detection of CD9 and CD63 expressions by Western blot; (c). After PKH67 labeling exosomes, they were added to breast cancer cells *in vitro*. From left to right, DAPI, PKH67, and combined figures (scale bar: 50 *μ*m).

After being extracted from the supernatant of MDA-MB-231 breast cancer cells cultured *in vitro*, the exosomes were observed under an electron microscope. The extracted exosomes displayed a disk-like shape in structure with diameters of 50-100 nm (Figure 1(a)). The tetraspanins, CD63 and CD9, have been widely used as markers for exosomes because of their accumulation in small extracellular vesicles compared to whole-cell lysates and the steady-state accumulation of CD63 in multivesicular body [25]. Western blot was used to detect exosomal marker proteins, and the expression levels of marker proteins CD9 and CD63 in exosomal proteins were markedly increased compared with the control group (total cell protein) (Figure 1(b)). The isolated exosomes were labeled with PKH67 and added to the cultured breast cancer cells *in vitro*. Fluorescence results indicated that exosomes could enter into breast cancer cells through integration (Figure 1(c)).

### 3.2. Regulation of the Exosomes on Migration, Invasion, and Colony Formation of Breast Cancer Cells

**Figure 2:**
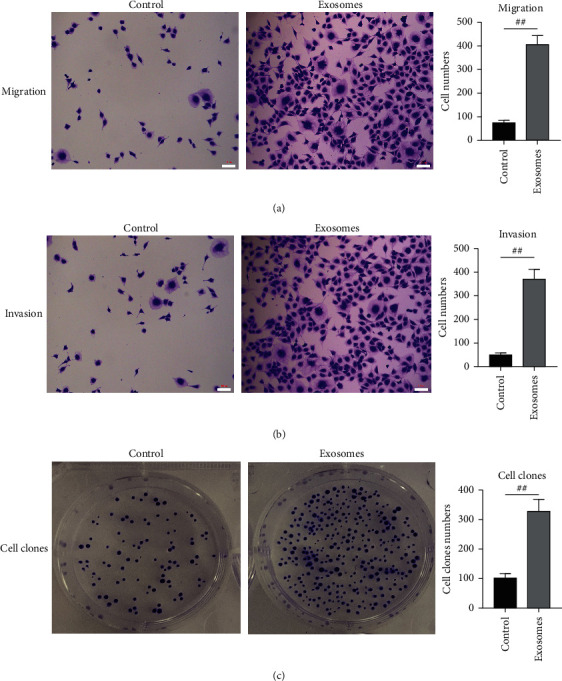
Regulation of the exosomes on migration, invasion, and colony formation of breast cancer cells. (a). Effect of exosomes on breast cancer cell migration. (b). Effect of exosomes on the invasion of breast cancer cells. (c). Effect of exosomes on colony formation of breast cancer cells. *N* = 3. Scale: 100 *μ*m. Data were shown as Mean ± SEM. ^##^*p* < 0.01. Treatment: exosomes. 20 *μ*L exosomes/mL cell medium; control, the control group was equal volume PBS.

When the exosomes of breast cancer cells MDA-MB-231 were added to cultured breast cancer cells, the number of migrated breast cancer cells in the exosomal treatment group was 408.30 ± 20.93, and that in the control group was 74.67 ± 5.48, indicating a substantial increase in the number of migrated breast cancer cells in the exosomal treatment group (Figure 2(a)). The number of invasive breast cancer cells in the exosomal treatment group was 372.00 ± 23.29, and that in the control group was 51.00 ± 4.62. The number of invasive cells in the exosomal treatment group was markedly increased (Figure 2(b)). The clone number of breast cancer cells in the exosomal treatment group was 328.30 ± 22.81, and that in the control group was 103.00 ± 8.39. The clone number of breast cancer cells in the exosomal treatment group was also greatly elevated (Figure 2(c)).

### 3.3. Regulation of NUMB in Breast Cancer Cells by Exosomes

**Figure 3:**
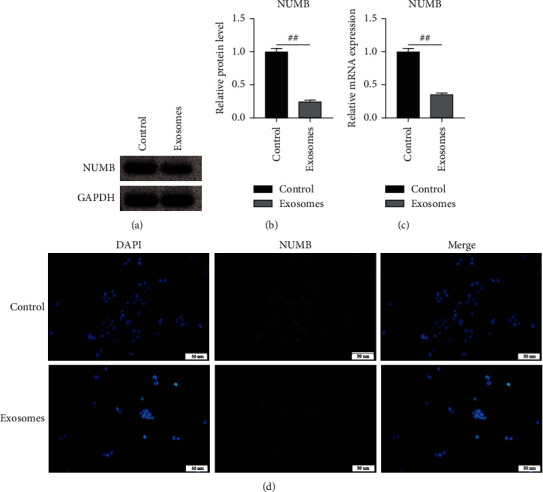
Regulation of NUMB by exosomes. (a). Detection of NUMB protein expression by Western blot; (b). Western blot grayscale statistics; (c). NUMB mRNA expression was detected by RT-PCR; (d). Detection of NUMB protein expression by immunofluorescence. *N* = 3. Data were shown as Mean ± SEM. ^##^*p* < 0.01. Treatment: exosomes. 20 *μ*L exosomes/mL cell medium; control, the control group was equal volume PBS.

**Figure 4:**
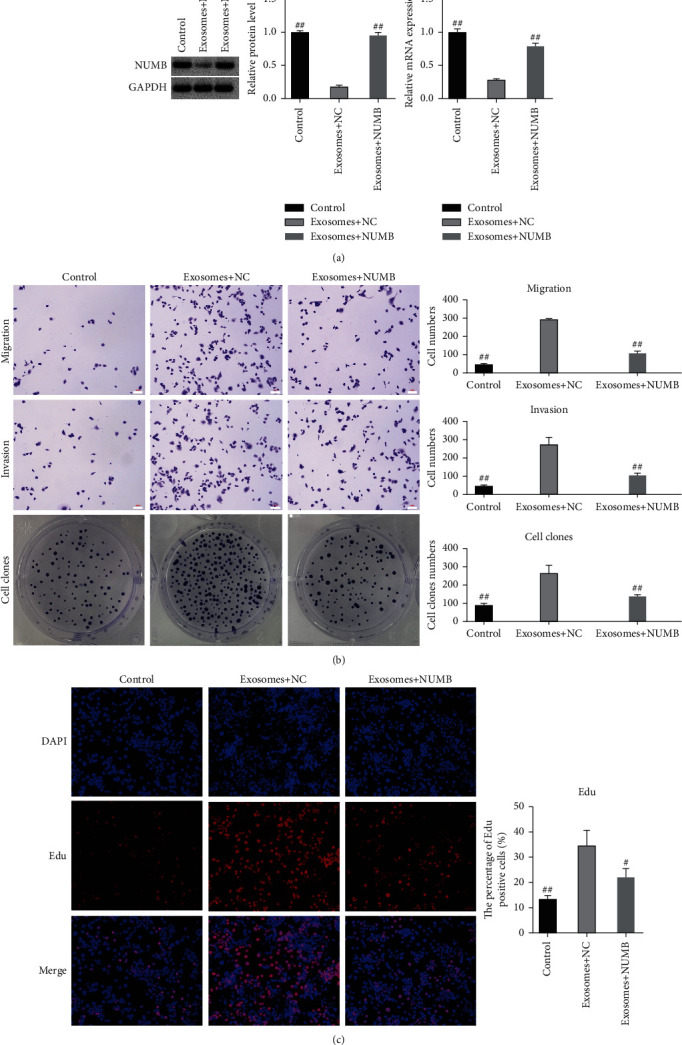
Verification test for NUMB recovery with overexpression. (a). NUMB expression in the blank control group, exosome+NC group, and exosome+NUMB group was detected by Western blot and qPCR. (b). Detection of cell migration, cell invasion, and cell colony formation. (c). EDU was used to detect cell proliferation. *N* = 3. Scale: 100 *μ*m. NC (negative control), add empty virus control group. Data were shown as Mean ± SEM. ^##^compared with the exosome+NC group, *p* < 0.01; ^#^compared with exosome+NC group, *p* < 0.05. Treatment: exosomes. 20 *μ*L exosomes/mL cell medium; control, the control group was equal volume PBS.

NUMB protein and mRNA expression levels in breast cancer cells treated with exosomes were sharply decreased (Figures 3(a)–3(d)), indicating that exosomes could inhibit NUMB expression in breast cancer cells. We subsequently performed a recovery validation assay for NUMB overexpression in exosome-treated breast cancer cells. NUMB overexpressed lentivirus and control null virus were added into exosomal-treated breast cancer cells to effectively restore the inhibitory effect of exosomes on NUMB expression (Figure 4(a)). Additionally, cell migration, invasion, and clone formation were detected compared with the exosome group (empty virus). NUMB overexpressed lentivirus could effectively reduce the invasion, migration, and proliferation of breast cancer cells (Figure 4(b)). NUMB overexpression markedly declined EDU positive cell ratio in breast cancer cells compared to the exosome group (empty virus), suggesting that NUMB could suppress the proliferation of breast cancer cells (Figure 4(c)).

## 4. Discussion

This present study isolated and identified exosomes from breast cancer cells and added the labeled exosomes to cultured breast cancer cells *in vitro*. The findings of this project indicated that exosomes could promote the migration, invasion of breast cancer cells, and the formation of cell clones. Meanwhile, it also demonstrated that exosomes could reduce NUMB expression levels in breast cancer cells. Furthermore, NUMB could inhibit proliferation, invasion, and migration of breast cancer cells by adding with overexpressed NUMB lentivirus.

Exosomes are recognized as nanoscale membrane vesicles produced by multiple cells. They exert a pivotal role in intercellular communication. In breast cancer, exosomes act as mediators of oncogenic information between local and systemic cells, carrying out the transfer and signal transduction of various bioactive molecules, namely, proteins and mRNAs. Meanwhile, they affect the progression of tumors [26]. Some research has indicated that exosomes of breast cancer cells can change the transcriptome of target cells and assist in transforming oncogenesis and forming tumors. Take the exosomes derived from MDA-MB-231 for an example, they are responsible for transforming normal breast epithelial cells (MCF10A) into tumor cells [27]. Furthermore, the exosomes from MDA-MB-231 can weaken the therapeutic effect of chemotherapeutics on breast cancer and enhance the chemoresistance of cancer cells [28]. Pan et al. also found that exosomes from MDA-MB-231 breast cancer cells promote angiogenesis of human umbilical vein endothelial cells [29]. In cultured cells *in vitro* and mouse models, these miRNAs containing tumor cell exosomes alter the transcriptome of recipient cells by inducing RNA relevant complex proteins. Pre-miRNA dicer, TAR RNA-binding protein 2, and argonaute-2 can be processed into mature miRNAs. Additionally, they play a local role in promoting tumor formation and proliferation and affecting distant cells in migration and invasion [30, 31]. When tumor cells manipulate the local microenvironment, tumor metastasis is available after conditions of cell invasion and growth are optimized. The process of adhesion is of great significance both in a variety of pathological states and tumor biology. Cell segregation links to exosome release in breast cancer cells. Exosomes have been identified to aggregate on the cell surface and implicated as a key factor in mediating the adhesive effect to extracellular matrix proteins [6–32]. This study revealed that by adding the exosomes extracted from MDA-MB-231 to cultured breast cancer cells *in vitro*, the capacities of cell migration and invasion and the number of clone formation could be substantially improved, which was consistent with previous research data. To sum up, these data obtained suggested that the exosomes obtained from breast cancer cells could enhance the ability of cell migration and trigger its migration that was in proportion to cell metastatic potential.

NUMB is known as an evolutionally conserved protein, and it exerts a key role in determining cell fates, maintaining polarity, cell migration, and endocytosis. NUMB also suppresses tumor progression, including the regulatory effects on the processes of physiological development [33]. It has been reported that NUMB can be downregulated in malignant pleural mesothelioma, salivary adenocarcinoma, breast cancer, esophageal squamous cell carcinoma, and nonsmall cell lung cancer [34]. It can interact with CDH1 and CDH2 and control appropriate position of relevant key adhesion molecules within cells, but the NUMB expression is downregulated, which disrupts cell-cell adhesion allowing cell migration. NUMB also participates in HGF-induced epithelial-mesenchymal transformation by regulating E-cadherin and cellular polar molecules PAR3, PAR6, and APKC [35, 36]. Furthermore, it is associated with p53 and Notch, and its interactions with MDM2 stabilize p53 expression in renal fibrosis and hepatocellular carcinoma, including breast cancer. This protein contributes to promoting ubiquitination and degradation of Notch1 intracellular domains thereby enabling the inhibition of Notch signaling. Taken together, NUMB loss in tumors may be involved in epithelial-mesenchymal transformation through dual mechanisms of p53 inhibition and Notch activation [35–37]. Total NUMB protein expression was significantly decreased in breast cancer cells treated with exosomes, whereas cell migration and invasion were increased. The findings of current research demonstrated that breast cancer exosomes might further promote the migration and invasion of breast cancer cells when the NUMB expression levels were reduced, and the molecular mechanism of their regulation needs to be further investigated. The limitation of this study is that it failed to further verify the regulation of breast cancer cell exosomes on breast cancer and the regulation of NUMB protein expression at the animal level. And it lacked high-throughput sequencing and more in-depth molecular mechanism research.

In further studies, we will conduct transcriptome and proteomic analysis on breast cancer exosomes to clarify the key components and the signal transduction mechanism of the key components, to lay a certain research foundation for revealing the regulatory role and molecular regulatory role of breast cancer exosomes as well as breast cancer treatment.

## 5. Conclusion

The breast cancer cell-derived exosomes could enter into breast cancer cells through integration and inhibit the NUMB expression, promote cell migration, invasion, and formation of cell clones in MDA-MB-231 breast cancer cell line.

## Data Availability

The data used to support the findings of this study are included within the article.
